# Using perturbations to identify the brain circuits underlying active vision

**DOI:** 10.1098/rstb.2014.0205

**Published:** 2015-09-19

**Authors:** Robert H. Wurtz

**Affiliations:** Laboratory of Sensorimotor Research, National Eye Institute, National Institutes of Health, Bethesda, MD 20892-4435, USA

**Keywords:** brain-circuits, behaviour, monkeys, lesions, inactivations, optogenetics

## Abstract

The visual and oculomotor systems in the brain have been studied extensively in the primate. Together, they can be regarded as a single brain system that underlies active vision—the normal vision that begins with visual processing in the retina and extends through the brain to the generation of eye movement by the brainstem. The system is probably one of the most thoroughly studied brain systems in the primate, and it offers an ideal opportunity to evaluate the advantages and disadvantages of the series of perturbation techniques that have been used to study it. The perturbations have been critical in moving from correlations between neuronal activity and behaviour closer to a causal relation between neuronal activity and behaviour. The same perturbation techniques have also been used to tease out neuronal circuits that are related to active vision that in turn are driving behaviour. The evolution of perturbation techniques includes ablation of both cortical and subcortical targets, punctate chemical lesions, reversible inactivations, electrical stimulation, and finally the expanding optogenetic techniques. The evolution of perturbation techniques has supported progressively stronger conclusions about what neuronal circuits in the brain underlie active vision and how the circuits themselves might be organized.

## 1. Introduction

Our vision and that of all primates is a continuous interaction between input from the retina to the brain and eye movements produced by the brain. Primate vision relies on input from the central region of the retina, the fovea, the retinal region that provides the highest spatial resolution. It is this high-resolution vision that is critical for examination of all objects of interest. This fovea in turn requires eye movements that direct it sequentially to different parts of the visual field to examine one object after another. For this purpose, we have a specific type of eye movement, the saccade, that rapidly redirects the fovea. These saccades occur at a high rate, frequently two or three per second, which provides new and detailed visual information to the brain at that same rate. Saccades also produce problems for vision: the image of an object lying on one part of the retina suddenly lies on another part after each saccade, and during the high-speed saccade the image from the retina is a blur. Brain mechanisms have developed to compensate for these displacements in ways only roughly understood [1]. This process of eye movement manipulated visual input has been termed active vision, and it is a functional amalgamation of visual processing and eye movement control. It is a complex system but it must be working well, given that primates have become the dominant vertebrate species on the planet.

**Figure 1.**
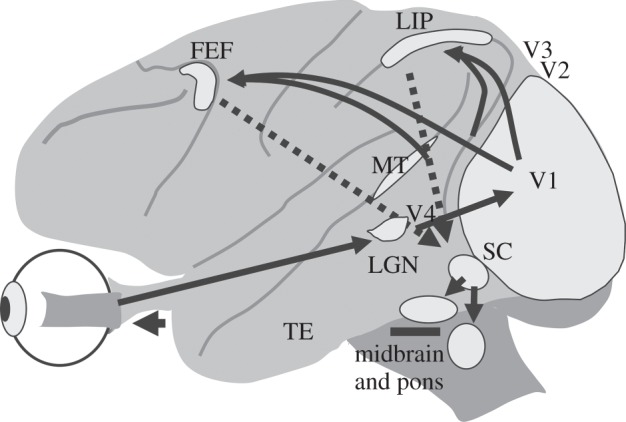
The brain circuits for visually guided saccades extend from cerebral cortex to the pons in the brain stem. This side view of the monkey brain shows that the circuit extends from retina to primary visual cortex (V1), then to extrastriate cortex, particularly to the lateral intraparietal (LIP) area and frontal eye field (FEF). From cortex, information reaches the superior colliculus (SC), and from there to brainstem oculomotor areas in midbrain and pons, and finally to the extraocular muscle motor neurons that project to the eye muscles to move the eye. This is a simplified brain circuit, and does not show a number of other circuits including those of the basal ganglia and the cerebellum. MT, middle temporal cortex; LGN, lateral geniculate nucleus; TE, anterior inferior temporal cortex.

What we want to understand is how circuits in the brain underlying the visual–oculomotor system are organized to produce this active vision, which is probably the most prominent and the most extensive sensory-motor system in the primate brain. It consists of systems comprising multiple types of visual processing and eye movements, but here we will consider only the visual and saccadic eye movement division of these systems. This saccadic system extends from the highest cognitive levels of the frontal and parietal cortex to the motor neurons in the brainstem. Figure 1 provides a global outline of the system. In addition to generating saccades, the system also provides information to other brain regions to inform them of the impending visual changes that result from the saccades [2].

## 2. Dissecting brain circuits for active vision: the role of perturbation

The challenge is to dissect out the brain circuits that are related to the integration of vision and eye movements from the myriad circuits in the brain. For example, how do we know that a neuron activated by a visual stimulus is in a circuit for object recognition, targeting the next saccade, or dilating the pupil? The primary method to identify these circuits is to perturb them to test whether and how alterations change behaviour. By circuit I mean a series of connected neurons, or a population of neurons, that are required to execute a given behaviour. I realize this is a glib definition, but it is consistent with the level of our current knowledge; few circuits in the brain have been worked out in detail so that evaluating the definition is part of the problem in studying ‘brain circuits'.

Examining a brain circuit and its relation to behaviour moves through a series of steps, though rarely in such a neat order as described here. The first step has been to determine the anatomical connections that might underlie a particular function. Many of the major pathways in the visual system have been known from the early twentieth century from anatomical studies and evoked visual potentials. Information about the oculomotor output became available in the middle of the twentieth century when neuronal activity studied in awake animals could be added to anatomical knowledge. Critical details are still lacking, but unfortunately anatomy has currently fallen out of funding favour, and most of the gaps are unlikely to be closed soon. Once connections are identified, the functional contribution to active vision can be considered, although on the basis of anatomy alone, these are largely guesses.

The second step is to correlate neuronal activity at different nodes of a putative neuronal circuit to a particular behaviour, from the visual input to the eye movement output, but with concentration on the vast processing in between. The earliest studies usually began with neurons of known anatomical connections on the input side (from retina though the lateral geniculate nucleus to visual cortex) and on the output side (from a few brainstem nuclei to motor neurons to eye muscles). The next step is estimating the function of these neurons, and here the prime method is correlating neuronal activity to visual behaviour, using both psychophysical measures of visual processing and motor performance measures of eye movements. This approach was the basis of the initial investigations of the visual system such as those of Kuffler [3] in the retina and Hubel and Wiesel [4,5] in the cortex of anaesthetized cats and monkeys, though of course the resulting behaviour was hypothesized not measured. The same correlation approach was later used by Wurtz in awake monkeys trained to hold their eyes steady for several seconds to allow analysis of neuronal receptive fields [6]. In the oculomotor system, the comparable initial correlations were between motor neurons in the oculomotor nuclei and saccadic eye movements [7–9]. The most difficult problem, however, has been relating the vast majority of the neurons that are neither at the input nor the output but that comprise the processing that lies between.

The third step is the most challenging: showing that a given visual–oculomotor activity is *necessary* for active vision and not some other function such as adjusting pupil size. This step is usually done by perturbing the system. We predict what should happen to behaviour when we either reduce or enhance the activity of a given element in a brain circuit. We then test the prediction by experimentally reducing or enhancing it to see whether the result is consistent with the prediction. Correlations hint at the functions of circuits, but they do not establish them. By perturbing the system the effect on behaviour can be assessed, and the element perturbed can be identified as one that does or does not contribute to the behaviour of interest. The sharper the prediction, the more precise the functional answer. Without this step of perturbation, we have only a catalogue of connections and a collection of correlations. Perturbation moves the understanding of neuronal activity from correlation closer to causality and has been the essential step in relating neuronal activity to active vision.

In this article, I will describe the series of perturbation approaches that have solidified our knowledge of the visual–oculomotor system underlying active vision in the monkey. The easiest way to describe the evolution of this approach is to describe the techniques in roughly historical order, and then illustrate them with example applications. As these are just examples, many will be from my own work, particularly those on the saccadic system. I trace not only the succession of techniques but what advances each provided and what the drawbacks were that led to the adoption of new techniques.

## 3. Ablations

Much of our initial knowledge of the visual–oculomotor functions resulted from observing the consequences of brain lesions. Humans who had suffered vascular accidents or trauma provided essentially all the information that was available. The limitation of these observations on humans was substantial; knowledge of lesion location within the brain was frequently lacking and only limited measures of behavioural deficits were performed. In the second half of the nineteenth century, this began to change as experimental animals came into use, mainly cats and dogs, but some monkeys as well.

An illustration of the importance of this new approach can be appreciated by recognizing one of the major early achievements of animal research: locating the primary visual cortex (V1). Ferrier in England had done ablations in the monkey cortex and concluded that V1 was located in the parietal lobe in the region of the angular gyrus. Monkeys with such lesions had a blank stare and ran into objects as they were led about the room. The conclusion was highly controversial because Munk in Berlin argued that lesions in the occipital cortex, rather than the parietal cortex, led to blindness. A spirited controversy followed, and as we now know, Munk was right and the possible reasons for Ferrier's error have been fully summarized by Glickstein [10]. The reason this controversy is important for our purposes is that it illustrates a potential pitfall of using the technique. The changes in an animal's behaviour had to be systematically analysed just as the ablations had to be systematically placed in the brain. The resolution of the visual cortex controversy was based largely on better analysis of the behaviour demonstrating blindness (including recovery from the lesion that we will address later). The quantitative measure of behaviour is as important to the perturbation method as is the placement of brain lesion, if not more so. The central importance of the measurement of behaviour will persist through all of the uses of perturbation that we consider.

The use of experimental animals in ablation experiments incorporated at least two anatomical advances: specific structures could be removed and the extent of the removal could be verified post-mortem, including histological verification. For cerebral cortex, the most frequent ablation technique has been subpial suction of the cortical grey matter which can be extended over substantial regions of cortex depending upon the skill and the perseverance of the surgeon. A frequent technique for subcortical ablations has been electrolytic lesion that largely kills cells by the heat generated by electric current passing though the brain. Here, the lesion was less predictable because current spreads and can expand a lesion in unexpected directions, as was the case with some early electrolytic lesions of the superior colliculus (SC) [11].

With the advent of neural recording, including evoked potentials, electroencephalography, and eventually single neurons, the lesions could be more accurately directed to those regions with known relations to a specific visuomotor behaviour. Furthermore, with the advent of imaging, a good estimate of the brain areas affected can be obtained while the subject is still alive.

A further example of the evolution of these techniques in the visuomotor system is the growth of knowledge on the organization and function of V1. The first information resulted from unplanned ablations: gunshot wounds studied following the Russo-Japanese war. By mapping visual field defects, a Japanese physician, Inowe, was able to establish an outline of the relation of different parts of the visual field to specific segments of visual cortex [12]. When experimental animals were used, the mapping could be refined by using evoked responses and later neuronal activity to precisely map the visual field in V1 of anaesthetized cats [13] and later in anaesthetized monkeys [14]. In the awake monkey, subpial suction of V1 led to deficits in detection of briefly flashed stimuli in the regions of the contralateral visual fields just as expected [15]. The monkey also could not make accurate saccades to targets in the contralateral fields, a deficit that was interpreted as ‘if you can't see it, you can't make a saccade to it’. Similar deficits in vision and in saccades were found in subsequent ablation experiments in V1 [16].

**Figure 2.**
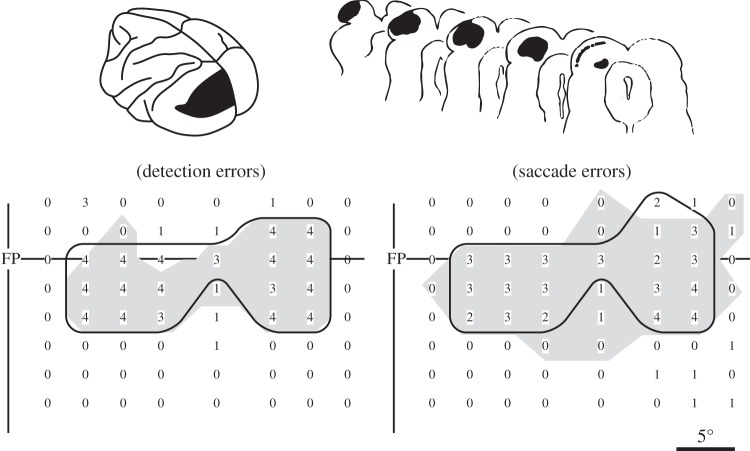
An illustration of the methods and the limits of ablation. A subpial suction ablation of V1 cortex (top left) produced blindness in a segment of the visual field (shaded area on the bottom maps indicates at least two errors in four trials) for both stimulus detection and saccades. Within a month, this blindness recovered, which illustrates that during that month both the lesion deficit and the changing state of recovery were affecting the behaviour being tested. Addition of an electrolytic lesion in SC (top right) produced a blind region in the visual field related to the SC ablation (area within the solid line on the bottom maps again indicating at least two errors on four trials). Where the two lesions overlapped, the blindness remained for the duration of the experiments (4–15 weeks). Adapted from Mohler & Wurtz [15].

What was less expected is a factor that is a major limitation of many ablation studies: both visual detection and saccade deficits gradually decrease, so that after a month [15] or two [16], spots that could not be detected just after the ablation now could be seen, and saccades could be made to these targets. As the removed area of visual cortex is still not there, this can only indicate that pathways other than the one through thalamus to V1 are able to provide visual information. This recovered ability might well be related to the phenomenon of blindsight in which human subjects with extensive damage to visual cortex can respond to stimuli in the ‘blind’ visual field [17]. What other brain regions provide the recovery after a cortical ablation is frequently difficult to determine, but in the case of recovery after ablation of V1, the structure that contributes heavily is well known: the SC. Mohler & Wurtz [15] found that damage from electrolytic lesions of the SC alone did not produce a visual detection deficit or more than a transient saccade deficit. But ablation of both V1 and SC led to complete blindness in the part of the visual field where the effect of the lesions overlapped—for both visual detection and saccadic accuracy (figure 2). This observation is the root of the hypothesis that what can be seen in blindsight (the ability for partial vision in an otherwise blind visual field) might be based in large part on what visual information can be derived from the SC via its pathways to cerebral cortex.

The major point to emphasize, however, is that recovery of sight in the monkey is a recovery that occurs over time after the ablation. So when the monkey's visuomotor ability continues to be studied after the ablation, two factors are actually being studied. The first is the loss of a function from the lesion, and the second is change in other brain areas that enable them to assume at least part of the function of the ablated brain. What is studied after an ablation is not a stationary process, but one that is constantly changing due the compensation mediated by other brain areas.

The interaction between brain areas after ablations is further illustrated by the finding that extending a single lesion to lesions of multiple brain areas need not create a greater deficit. The classic illustration of this is the Sprague effect. Removal of occipito-temporal cortex of a cat leads to reduced orientation to the contralateral visual field as expected. The surprise is that removal of the SC contralateral to the cortical ablation restores the orientation to the visual field that had appeared to be blind. Vision was restored by taking out additional brain [18]. The explanation of this has been pursued over the last half century and has revealed the interactions between multiple brain areas, as has been recently summarized [19].

The significance of cortical ablations and electrolytic lesions is that they provided the first tests of the relation of cortical and subcortical areas to visuomotor activity. The original method for establishing the relation of a brain structure to behaviour is still a cornerstone technique in neuroscience in large part because of subsequent refinements. A major drawback is that as behaviour is studied following the ablations, it is a combination of the lesion deficit and the constantly changing recovery from that deficit.

## 4. Chemical lesions

Injection of neuro-active chemicals to produce a lesion addresses the problems of the interaction between lesion deficit and its recovery by measuring behaviour before there is time for recovery. The injections are small, usually done with a microliter syringe, and can be made on one day and the behavioural effect tested on the next day. The day wait follows from the fact that the neuro-active chemicals are killing neurons, and their effect is usually not instantaneous. Waiting until the process is closer to being complete avoids studying behaviour when there might be transient activity, such as a hyperactivity preceding neuron death. In addition to behavioural measurement soon after the lesion, a significant advantage of the chemical lesion over the surgical or electrolytic ones, is that the injection can be precisely directed by first locating the target area with neuronal recording. The precision can be increased further by verifying the location of the injection by concurrent recording from an electrode attached to the injection syringe [20]. Finally, the chemical lesions leave a record of their location for future histology. This location also is relatively easy to determine because the integrity of the layered or nuclear structure is maintained. Only the damaged neurons are removed from the structure.

**Figure 3.**
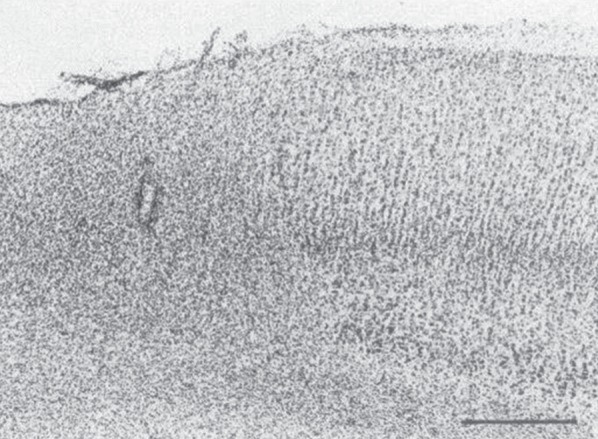
An advantage of chemical lesions: histological verification with the basic brain structure still visible. The chemical lesion was produced by ibotenic acid injected into area MT. The brain section is parasagittal, stained with cresyl violet for cell bodies and shows the ventral portion of the superior temporal sulcus. Dorsal is upward and anterior is to the right. Cortex on the left around the electrode track is grossly disrupted: there is a pronounced loss of neuronal cell bodies and massive gliosis. Cortex to the right of the injection area exhibits the normal columnar organization of cell bodies and laminar structure. The calibration is 500 um. Adapted from Newsome *et al.* [21].

An example of such a chemical lesion, one produced by ibotenic acid, comes from injections into the cerebral cortex, specifically the motion areas in the middle temporal cortex (MT) by Newsome *et al*. [21]. The injection affected a limited part of the visual field and the behavioural purpose was to test whether MT provided the directional motion information required to guide smooth pursuit. It did. The monkey's initial saccade to the moving pursuit target did not adjust for the motion of the target and the initial pursuit eye speed that results from visual motion input was reduced by the ibotenic acid lesion. The punctate lesion demonstrated the dependence of pursuit movement on the motion processing in one extrastriate area, MT. As in the lesion experiments we have considered in V1 and MT, the behavioural deficits recovered. Within about a week, the pursuit had recovered; the best tests of the deficit were on the day after the chemical lesion. The site of the injection was clearly determinable from the subsequent histology (figure 3). This example emphasizes an added advantage of a chemical lesion; the adjacent structure beyond the area of damage continues to show the cortical lamination in MT. With that structure intact, the extent of damage to specific layers can be assessed.

In net, the advantage of chemical lesions is the minimization of any recovery before a behavioural measurement is made, and the precise localization and the histological verification of the site of neuronal removal. The major disadvantage is that the lesion persists and is present in all subsequent experiments on the particular monkey. An additional disadvantage is the time it may take for the lesion effect to develop and so, in principle, some recovery of function could occur before the injection effect is measured.

## 5. Reversible inactivations

**Figure 4.**
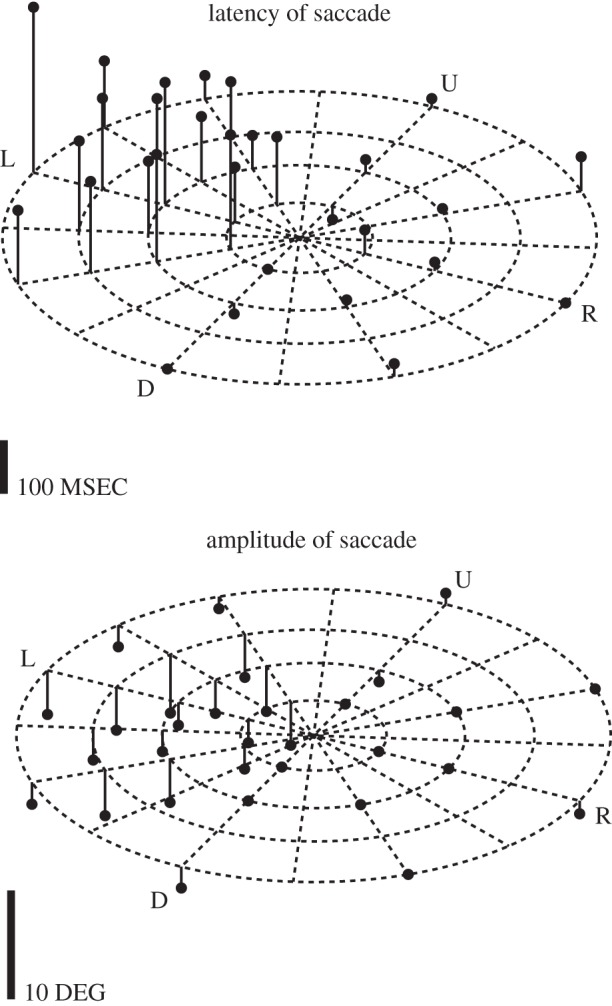
An advantage of reversible inactivation; behaviour can be tested immediately after the brain circuit is disrupted. The illustration shows any change in the latency and amplitude of saccades to visual targets across the visual field after muscimol inactivation of saccade-related neurons in the right SC. Eccentricities of dashed circles are 5, 10, 15, 20°—R, L, U and D are right, left, up and down, respectively. Only change in values for latency (top) and amplitude (bottom) following inactivation are shown: up is an increase and down is a decrease. The increase in the latency of the saccade (top) and the decrease in amplitude of the saccade (bottom) are limited to targets in the left (contralateral) visual field. Each point is the average of two trials. The deficits were clear when measured about an hour after the injection whereas with electrolytic lesions the tests that showed little deficits were done days after the lesions. Adapted from Hikosaka & Wurtz [23].

The problems of permanent damage to the animal with a chemical lesion are solved by the use of reversible inactivation. Injections using such agents as transmitter agonists or antagonists (for example muscimol or bicuculine for GABA**_A_** receptors) produce reversible inactivations so that the effect on behaviour can be determined immediately. In addition, complete recovery within a day or so leaves an intact monkey (except for any damage from recording electrodes or the injection syringe). The first place where the efficacy of these reversible lesions was used in the visuomotor system was in the SC with the behavioural test being its effect on saccade generation. The original electrolytic lesions of SC by Wurtz & Goldberg [22] produced surprisingly limited deficits, mainly a consistent increase in saccade latency and a transient increase in variability of saccade amplitudes. This was a surprise because the injections were made in the midst of neurons that discharged before saccades, neurons that were arranged in an orderly map within the SC to produce the vectors required for saccade generation. The role of the SC in saccade generation was reassessed when Hikosaka and Wurtz made reversible lesions in SC that were induced by microlitre injections of muscimol [23]. The use of muscimol was inspired by the previous findings of a GABAergic projection from substantia nigra to SC [24]; the SC neurons must therefore have the appropriate receptors. It was also one of the first experiments in the visuomotor system that used reversible inactivation in an awake monkey. In the SC, the inactivation produced the previously seen increases in saccade latency but also large changes in peak saccadic velocity and in the amplitude and direction of saccades (figure 4), particularly to target locations that had to be remembered. The interpretation of this far greater effect by inactivation than by lesion was that the deficits with muscimol were tested within minutes of the inactivation rather than after a lapse of days for electrolytic lesions. *Activation* of neurons was also achieved by using the GABA**_A_** antagonist, bicuculine, that produced irrepressible saccades to one part of the visual field even though the monkey was being rewarded for maintaining visual fixation. Both a decrease and an increase in activity were produced by different drugs acting on the same receptor.

It is worth noting that the GABA**_A_** agonists and antagonists (muscimol and bicuculine) seem to act on the input to the SC neurons rather than directly on the output of the neurons and probably not on fibres of passage; the threshold for eliciting saccades with electrical stimulation of the neurons affected by these GABA**_A_** receptor drugs did not increase. By contrast, use of an anaesthetic such as lidocaine did raise the threshold [25] indicating an effect on the output axons. The anaesthetic also affects any fibres of passage, whereas muscimol probably does not, and lasts minutes not hours.

Reversible inactivations in cerebral cortex are generally more difficult than those in subcortical areas such as those described for the SC because the area of cortex modified must be larger and across a relatively thin sheet of cortex to effectively alter behaviour. One solution is to make multiple injections spaced so that there is overlap between the separate injections. Another approach is to use the cooling of neurons to suppress their activity, which is obviously reversible [26,27]. Using this technique, larger areas of cortex can be inactivated than by localized injections.

The general point of the reversible inactivations is that they change behaviour almost immediately after an injection so that compensation for any deficit is minimized. Inactivation reveals the deficit, not the compensation. The very recovery we value so highly because we still have an intact trained monkey at the end of the experiment also unfortunately has the disadvantage of leaving little indication of the location and extent of the inactivated area. Determination of the inactivation location requires marking lesions near the injection site, and evaluating the spread of the injection requires comparing injection effects across a series of injection sites.

## 6. Electrical stimulation

The perturbations considered so far usually have been designed to test the contribution of a particular population of neurons to a specific behaviour by removing neurons from a brain circuit. The opposite approach is to enhance the output of a circuit by injecting a signal into it at a given time and a given place in the circuit. A few neuro-active chemicals (such as bicuculine) can provide the selective place activation but not the timing; once the chemical is in the brain, it continues to act at least for minutes. In fact, one of the earliest stimulation experiments on the SC was done by Apter in 1945 [28]. Using strychnine to stimulate the surface of the SC, she activated neurons and determined the representation of the visual field across the surface of the SC. Electrical stimulation, in contrast to chemical stimulation, provides added temporal control, it has been widely used for perturbing vision and evoking eye movements. The stimulus can be turned on and off rapidly, and pulsed to approximate a normal train of neuronal impulses. It has been the major technique used to provide temporal control of neuronal activity. The major drawback is that the electrical stimulation probably activates neurons that normally are never activated together, and this might generate an ambiguity at downstream targets of the stimulated area.

**Figure 5.**
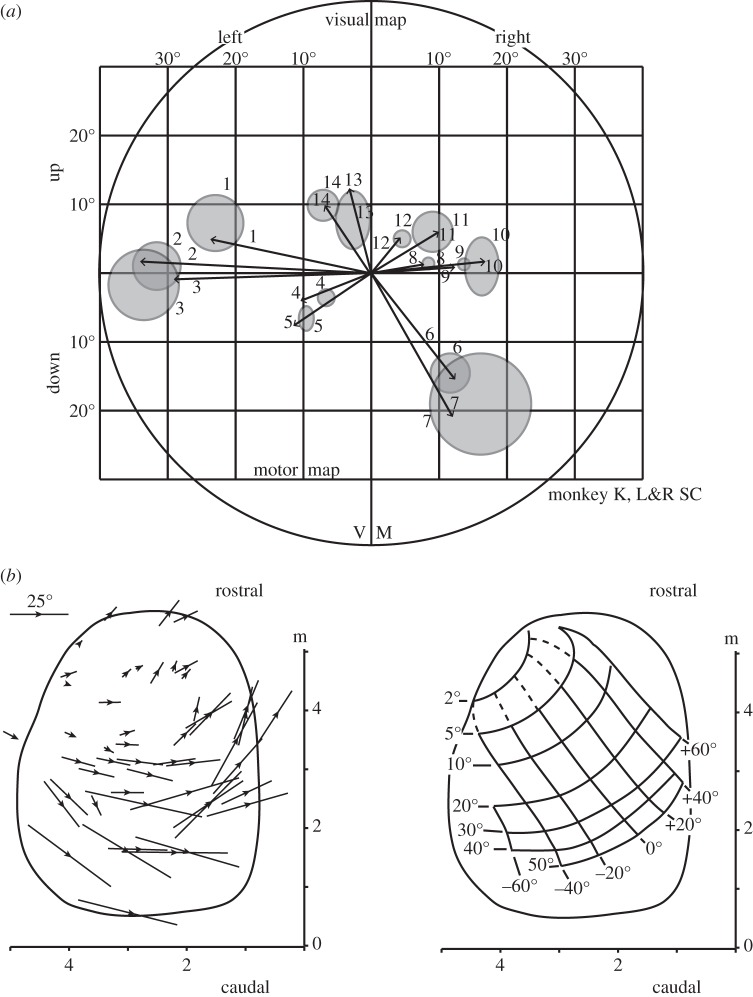
Electrical stimulation used to establish the relation between the location of neurons in the monkey SC and the saccades evoked by stimulation of the SC. (*a*) Comparison of SC visual fields (grey circles determined by visual neuron recording) and the vectors of saccades (arrows generated by stimulating at the site in the SC where the neurons with the visual receptive fields were located). The length of the arrow at each of the 14 sites represents the mean length of 8–14 stimulation-elicited saccades; the direction of each arrow represents the mean direction of saccades. The overlap between saccade ends and receptive fields is compelling. Adapted from [32]. (*b*) Determining whether there is an orderly map of amplitude and directions of saccades in the SC using saccades evoked by electrical stimulation. On the left are arrows indicating the amplitude and direction of saccades evoked by electrical stimulation at 42 points in the right SC. On the right are the smoothed contours of amplitudes from 2° to 50° and directions from −60° to +60° used to produce the standard map of the monkey SC. Adapted from [35].

Early examples of the use of stimulation can again be found in the SC. After neurons that discharged before saccades had been discovered [29], the issue was whether these neurons contributed to the generation of a saccade or were conveying a corollary of the saccade [30,31]. By electrically stimulating these neurons, Schiller & Stryker [32] were able to show that the neurons were driving saccades (figure 5*a*). With SC stimulation, saccades were directed to the same part of the visual field represented by the visual receptive fields of the stimulated neurons. The question of the generation of a corollary discharge by the SC, in addition to saccade generation was simply not asked by investigators at the time. That question was first addressed much later first by Sparks and co-workers [33] and then by Sommer & Wurtz [34].

One of the most striking uses of SC stimulation was by Robinson [35] who systematically stimulated at different points within the SC (figure 5*b*). From the direction and amplitude of the evoked saccades, he was able to derive the orderly map of the representation saccades in the primate SC. The map has become an icon in the field, and has consistently been confirmed and elaborated by other investigators [36].

Another frequent use of electrical stimulation has been to test the connections of one set of neurons to another set within a putative neuronal circuit—a key step in trying to identify circuits in the brain. This is a classic physiological technique [37,38], but its application to identifying circuits in awake monkeys is relatively recent, particularly in the visuomotor system [34]. The goal in using the technique is to determine the inputs to neurons by determining whether given source neurons provide input to a set of target neurons (using orthodromic stimulation of the source neurons and recording the action potentials in the target neurons) and whether that group of neurons is the source of input to the next set of target neurons presumed to lie in the circuit (using antidromic stimulation of these presumed target neurons and recording the action potentials in the previous set of neurons). The advantage of the technique is that it moves the anatomical description of a presumed circuit to the reality of an identified circuit. The major disadvantage of the technique is that it is difficult to execute: there is no conclusion from negative results. In order to draw any conclusion about either orthodromic or antidromic connections, the stimulation must activate neurons. Lack of activation tells nothing because the failure could be related to multiple causes. The technique has been most successful when both the source neurons and the target neurons fall on a retinotopic map and the recording points for both source and target locations are in the same part of the map. Slight deviations in the position of the stimulation points produce nothing but unbearable frustration. Examples of the successful use of the technique is in establishing the pathway from the SC movement neurons through the medial dorsal nucleus of thalamus to frontal eye field neurons [34] and from SC visual neurons through the inferior pulvinar to MT in parietal cortex [39,40]. The technique is a powerful one, but it is probably worth the considerable effort primarily when attempting to establishing the functional connectivity needed to establish a brain circuit. This is a case where an optogenetic approach (considered below) might produce critical simplifications. Substituting light stimulation for electrical stimulation might allow light to cover a larger area, facilitate adequate stimulation alignment, and even permit stimulating only a subset of neurons that have had a light sensitive construct placed in their membranes.

In summary, electrical stimulation has been one of the most widely used methods to establish the relation of neurons to behaviour in the visual–oculomotor system and to establish the relation between neurons within hypothesized brain circuits. Its strength is the precise timing of the stimulation. Its drawback is that it is likely to be activating groups of neurons that are rarely associated in normal physiological functioning. This problem is a particularly confounding one for electrical stimulation, but it in fact applies in varying degrees to all of the perturbation techniques considered.

## 7. Perturbing neurons with optogenetics

The developing approach to perturbation referred to as optogenetics has the potential to speed the obsolescence of the perturbation methods summarized so far. Optogenetics provides a method to turn on or turn off neurons for brief periods by activating a photosensitive construct inserted into the neuronal membrane. The construct is usually fused to a fluorescent protein such as GFP in an adenoassociated virus (AAV) with an appropriate promoter. The virus is injected into a brain region and the virus installs the photosensitive construct (for an ion pump or ion channel) into the neuronal membrane. Shining a light onto the neuron, at a wavelength specific to the construct, either depolarizes or hyperpolarizes the membrane depending on the construct inserted into the membrane. Excitation of neurons is produced, for example, by a channelrhodopsin, which when activated opens a channel through the membrane to produce excitation. Inhibition is produced, for example, by ArchT that acts by pumping protons across the membrane to produce inhibition. As this is written, the expansion and refinements of the technique are continuing and the technique is a promising one rather than a fully developed one. Stay tuned.

**Figure 6.**
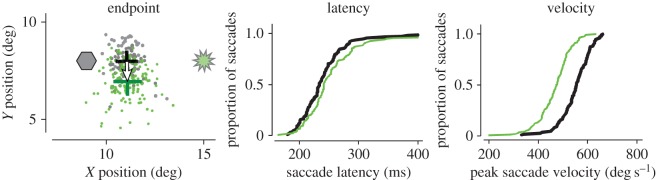
Tests of the behavioural effects of optogenetic inactivation of SC saccade-related neurons previously transfected with Arch-T, which should inactivate the SC neurons in the presence of green laser light. Saccades were made to a visual target with and without laser stimulation. On the left, saccade endpoints are shown without (grey) and with (green) laser stimulation. Laser light was introduced into the intermediate layers of the SC on randomly interleaved trials at a point related to the visual field position indicated by neuronal recording. Green and black crosses indicate the mean saccade endpoints (±1 s.e.m.) with and without light, respectively. The grey hexagon is the site of the injection, and the sunburst is the location of the green light source, both displayed at the sites in the SC indicated by neuronal activity from an electrode attached to the injection pipette or to the stimulating optic fibre. In the centre and right are the average differences in saccade latency and saccade velocity respectively. Adapted from Cavanaugh *et al.* [42].

In the visuomotor system of the monkey, two initial tests illustrate the potential power of the technique. In the visual system, activation of neurons in V1 led monkeys to shift their gaze towards the receptive field of neurons that were optically activated [41]. The activation was driven by channel-rhodopsin-2-expressing neurons. Light for activation was provided by an optic fibre on the surface of the dura. In the SC, the technique was tested on the saccade-related neurons to test whether their optogenetic inactivation would produce deficits in saccade generation. An AAV virus carrying ArchT was injected into the intermediate layers of the SC where the saccade-related neurons are located [42]. An optic fibre with an attached recording electrode for localization (an optrode as opposed to an injectrode) was inserted into the saccade-related neurons. As ArchT hyperpolarizes neurons, it should act on saccades like the reversible inactivations produced by muscimol. As illustrated by the sample inactivation in figure 6, the change in the saccades with inhibition is what we would expect from knowledge of the previous muscimol experiments [23,43]: the amplitude of the saccade was reduced or shifted, the latency was increased and the peak velocity was reduced. So the optogenetic perturbation produced the same effects as muscimol, but the effects were substantially smaller. Size of effect is important because of the requirement in most cases that many neurons must be altered to produce clear changes in behaviour. In hindsight, the small size of the behavioural change is not surprising because ArchT produces membrane potential changes by using a proton pump rather than the more robust opening of a channel, a problem that is being addressed [44]. A related issue is whether the volume of neurons sensitive to light can be sufficiently increased so that adequate light reaches enough neurons to modify behaviour. This is particularly important in deep structures where the size and number of optic fibres for light delivery is limited.

Even with the limitations of the present techniques, however, there are several salient advantages to the optogenetic method. First and most important is the interleaving of control and experimental trials, trials without the light and those with. This allows comparison of experimental and control trials with only seconds between them compared with chemical inactivations in which control trials frequently are in separate blocks and an hour away from the experimental trials at best. It is the ultimate elimination of any ambiguous results due to recovery! More importantly, it avoids the inevitable effects of changes in monkey performance as the testing session progresses, an effect that differentially alters whatever block of trials comes last. Second, optogenetic inactivation over a series of trials is nearly constant compared with the drug injections that are always changing due to the spread of the drug and its continuing degradation. Third, localized inactivation can be moved about to different locations within the region of transfected neurons simply by moving the optrode. Within what must be a gradient of transfection, the area activated or inactivated can be small enough to produce precise effects [42]. Finally, given that the virus introduces the constructs into the membranes of the axons as well as the cell bodies, the projections from one area to another also should be not only visible but subject to selective activation and inactivation. The probability is that future advances will make the activation and inactivation of specific neuron types and selected neuronal connections possible, in some cases even in monkeys.

The net point is that the optogenetic technique is a more powerful technique for perturbing a system within the brain than any of the current techniques summarized here. All techniques arrive on the scene with parameters to be tested and limitations to be established and optogenetics is no exception. But these issues will be resolved just as they have been for the other perturbation techniques. Even if only some of these advances in the technique occur, optogenetic perturbations will certainly revolutionize the study of systems in the brain, at least for the visuomotor systems in the monkey.

## 8. Conclusion

Perturbation of the visual–oculomotor system underlying our active vision has been one of the key methods for establishing the relation of neurons to behaviour. Many of the same techniques have been used as well to determine the relation between neurons within the brain as we attempt to identify the neuronal circuits in the brain that underlie the visual–motor behaviour. Without the perturbation step, we have just a correlation between neurons and behaviour or between one set of neurons and another. With perturbation, we can establish that given neurons are causally related to a given behaviour or to succeeding neuronal activity. Perturbation is a key part of what makes systems neuroscience an experimental science rather than an observational one.

The perturbation techniques have been a major contributor to making the visual–oculomotor system one of the best understood in the brain. Reviewing the prominent methods of perturbation used over the last century provides a snapshot of the evolution of techniques, from both cortical and subcortical ablations, to chemical lesions, to reversible inactivations, and finally to electrical stimulation. The story has a happy future in that the optogenetic techniques on the horizon promise to expand the perturbation method to more easily dissect out neuronal circuits within the brain and relate them to specific visual–motor behaviour.
